# Overcoming challenges in metagenomic AMR surveillance with nanopore sequencing: a case study on fluoroquinolone resistance

**DOI:** 10.3389/fmicb.2025.1614301

**Published:** 2025-07-23

**Authors:** Bram Bloemen, Mathieu Gand, Moniek Ringenier, Bert Bogaerts, Kevin Vanneste, Kathleen Marchal, Nancy H. C. Roosens, Jeroen Dewulf, Filip Boyen, Sigrid C. J. De Keersmaecker

**Affiliations:** ^1^Transversal Activities in Applied Genomics, Sciensano, Elsene, Belgium; ^2^Department of Plant Biotechnology and Bioinformatics, Ghent University, Zwijnaarde, Belgium; ^3^Department of Internal Medicine, Reproduction and population medicine, Faculty of Veterinary Medicine, Ghent University, Merelbeke, Belgium; ^4^Department of Information Technology, Ghent University, Zwijnaarde, Belgium; ^5^Department of Pathobiology, Pharmacology and Zoological Medicine, Faculty of Veterinary Medicine, Ghent University, Merelbeke, Belgium

**Keywords:** metagenomic sequencing, antimicrobial resistance (AMR), nanopore sequencing, DNA methylation, strain-resolved metagenomics, plasmid host prediction

## Abstract

**Introduction:**

Antimicrobial resistance is an alarming public health problem, and comprehensive surveillance across environments is required to reduce its impact. Phenotypic testing and whole-genome sequencing of isolates are efficient, but culture-free approaches like metagenomic sequencing potentially allow for broader investigation of resistance gene occurrence, evolution and spread. However, technical challenges such as difficulties in associating antimicrobial resistance genes with their bacterial hosts and the collapse of strain-level variation during metagenome assembly, hinder its implementation.

**Methods:**

To illustrate how these challenges can be overcome, we applied Oxford Nanopore Technologies long-read metagenomic sequencing and novel bioinformatic methods to a case study focused on fluoroquinolone resistance in chicken fecal samples.

**Results:**

We demonstrate plasmid-host linking based on detecting common DNA methylation signatures. Additionally, we use new bioinformatic approaches for strain haplotyping, enabling phylogenomic comparison and uncovering fluoroquinolone resistance determining point mutations in metagenomic datasets.

**Discussion:**

We leverage long-read sequencing, including DNA methylation profiling and strain-level haplotyping, to identify antimicrobial resistance gene hosts, link plasmids to their bacterial carriers, and detect resistance-associated point mutations. Although some limitations remain, our work demonstrates how these improvements in metagenomic sequencing can enhance antimicrobial resistance surveillance.

## 1 Introduction

Antimicrobial resistance (AMR) is an alarming global health challenge and is estimated to lead to around 1.91 million deaths annually by 2050 if no action is taken, according to a recent forecast (GBD 2021 Antimicrobial Resistance Collaborators et al., 2024). Effective responses require both monitoring of antimicrobial usage and surveillance of resistant organisms, and this across human, veterinary, agricultural and natural environments. While conventional AMR surveillance is performed through isolation and phenotypic testing of bacteria to track AMR trends for predefined panels of antibiotics (BELMAP., 2024), genotypic investigation is vital for detailed insights in resistance mechanisms, AMR gene (ARG) transfer and co-occurrence (Anjum et al., 2017; Djordjevic et al., 2024). Currently, short-read whole-genome sequencing (WGS) of isolates is widely used for this purpose, and reliably predicts resistance phenotypes (Zhao et al., 2016; Feldgarden et al., 2019). However, short reads struggle to resolve repetitive regions, a limiting factor in the investigation of plasmids and other mobile genetic elements (MGEs) that can play a role in the transfer of ARGs across bacterial species and their environments (Maguire et al., 2020; Djordjevic et al., 2024). Advances in long-read sequencing, such as Oxford Nanopore Technologies (ONT) R10 flow cells, V14 chemistry, and improvements in basecalling accuracy, now enable complete and high-quality assembly of bacterial genomes and plasmids using long-reads only (Sereika et al., 2022; Agustinho et al., 2024; Sanderson et al., 2024).

While WGS of isolates is efficient for monitoring AMR, there are several limitations: First, WGS is typically only performed on a limited number of species subjected to official control. Second, not all bacteria can be isolated, but even when isolation is successful, culturing and selection further restrict the scope of species and resistances that can be investigated. Finally, only a small fraction of the bacterial population in a sample is isolated and sequenced. This risks missing important isolates, species, and resistance mechanisms, creating large blind spots. Metagenomic sequencing can potentially overcome these issues by sampling all genetic material, revealing a larger diversity of ARGs (i.e., the resistome) and their potential bacterial hosts in a sample. Typically, bioinformatic methods investigate the resistome in a metagenomic dataset either directly from raw sequencing reads (i.e., read-based approaches), or by first assembling the reads into larger contigs (i.e., assembly-based approaches) (Djordjevic et al., 2024). Read-based approaches rapidly detect ARGs by skipping the computationally intensive assembly step, but may suffer from low taxonomic precision (Bloemen et al., 2023; Van Uffelen et al., 2024; Chen et al., 2025) and provide limited or no information on the genomic context of the detected ARGs (Djordjevic et al., 2024). Furthermore, reliably detecting resistance-associated point mutations in single reads is challenging due to sequencing errors, especially with long-read technologies, although their read accuracy is continuously improving (Chen et al., 2025). Assembly-based approaches assemble reads into larger contigs that can be grouped into bins and metagenome-assembled genomes (MAGs) based on features such as coverage, sequence composition or assembly graph information (Agustinho et al., 2024). Additionally, by aggregating information from multiple reads, more accurate consensus genomes can be generated, while the increased length of contigs offer higher taxonomic resolution and more genetic context on ARGs, enabling linking them to their host replicon. In this approach, long reads generate more contiguous assemblies than short reads, particularly around ARGs and for plasmids (Yorki et al., 2023; Abramova et al., 2024). However, as assembly requires sufficient coverage (typically 3x or more), ARGs at low abundance can be missed (Yorki et al., 2023; Chen et al., 2025). Leveraging the strengths of both read- and assembly-based approaches could potentially maximize ARG detection and allow for more in-depth characterization of their hosts replicons, though frequently, only one method is used.

Metagenomic sequencing allows to probe all ARGs present in a sample, and can reveal their genetic context when assembly is successful. However, linking a plasmid carrying an ARG to its bacterial host based solely on nucleotide sequence remains challenging. Advances in long-read sequencing of native DNA allows for detection of DNA modifications such as N4-methylcytosine (4mC), 5-methylcytosine (5mC), and N6-methyladenine (6mA). In previous studies, this has been used to group plasmids with their hosts based on common methylation patterns (Beaulaurier et al., 2018; Tourancheau et al., 2021). However, with ONT this required establishing a non-methylated baseline signal by sequencing a second methylation-free library, while Pacific Biosciences sequencing has low sensitivity for 5mC motifs (Agustinho et al., 2024; Heidelbach et al., 2024; Soto-Serrano et al., 2024). Recent developments in ONT technology addresses these issues by detecting 4mC, 5mC and 6mA DNA-modifications in native sequencing reads from a single library, and new bioinformatic tools such as MicrobeMod (Crits-Christoph et al., 2023) and NanoMotif (Heidelbach et al., 2024) can use this information for detecting methylation motifs. NanoMotif also applies this information for metagenomic bin improvement and including plasmids in a host bin (Heidelbach et al., 2024). However, this approach has not yet been applied specifically to metagenomic AMR surveillance.

Another remaining issue in metagenomics is the recovery of strain-level genomes and genetic variation, as assemblies typically convolute genetic variation into a single consensus sequence when multiple strains of the same species are present (Nicholls et al., 2021; Agustinho et al., 2024). In the case of AMR, this consensus might mask low-frequency single nucleotide polymorphisms (SNPs) associated with resistance, resulting in undetected AMR genotypes, as some might be caused by point mutations. Furthermore, tracing of outbreak origins often requires genome-wide comparison of bacterial strains. Combining outbreak investigation with AMR surveillance provides a comprehensive picture of pathogen spread, resistance evolution, and public health risks, allowing for faster and more effective interventions. Using metagenomics instead of isolate WGS for this purpose presents challenges as it requires to recover genetic variation that co-occurs within a strain, commonly referred to as *haplotyping* or *phasing*. While short reads accurately detect variants between strains, long-read metagenomics can generate more contiguous haplotypes, and recent tools have been developed to recover strain genomes (Kazantseva et al., 2024; Shaw et al., 2024). However, these tools have yet to be applied in the context of AMR surveillance, e.g., for strain-level phylogenetic investigation and AMR profiling directly from metagenomic data.

In this study, we applied novel long-read metagenomic methods to detect fluoroquinolone (FQ) resistance in chicken fecal samples as study case. FQs are categorized as highest priority critically important antimicrobials by the WHO, based on evidence of FQ resistant pathogens being transmitted to humans from non-human sources (WHO, 2024). Furthermore, FQ resistance is an interesting proof-of-concept, as both plasmid-mediated ARGs and chromosomal mutations play a role in acquired resistance. For example, FQ resistance genes such as *qnrA, qnrB, qnrS* and *oqxAB* are often located on plasmids or MGEs that are difficult to assign to a host strain using metagenomics data. Additionally, the predominant FQ resistance mechanism is based on mutations in the coding sequence of the *gyrA* and/or *parC* genes, requiring accurate SNP detection for identification (Bush et al., 2020; Kherroubi et al., 2024). Both plasmid-mediated and SNP-based FQ resistance genes are frequently detected in feces of chickens in Belgian broiler farms, making such samples ideal test cases to detect both resistance determinants in real-life conditions (Ringenier et al., under review)^1^. Using this case study, we addressed several current challenges in metagenomic AMR surveillance using ONT sequencing. First, we took advantage of long reads by detecting ARGs and their immediate genetic surroundings in a read-based approach, and used long-read assemblies for a broader view. Next, we applied ONT-based methylation calling and motif detection using Nanomotif (Heidelbach et al., 2024) to link an ARG-carrying plasmid to its host. Finally, we used haplotype phasing to uncover strain-level SNPs involved in FQ resistance, which were not detected at the MAG level. The applied phased reads were then used for a phylogenomic comparison between metagenomics and isolate WGS data. By addressing these challenges, we aimed to demonstrate how long-read metagenomics can be used for improved AMR surveillance.

## 2 Materials and methods

### 2.1 Sample collection, processing, and DNA extraction

One chicken (*Gallus gallus*) fecal sample per farm was taken from fresh droppings on each of two Belgian farms, A and B, and are hereby referred to as sample A and B, respectively. The fecal samples were collected using DNA/RNA Shield Fecal Collection Tubes (R1101; Zymo Research, Irvine, United States). Approximately 1 gram of fecal sample was taken and submerged in 9 ml of DNA/RNA shield. Samples were then stored at −80°C until further processing. To extract DNA, 100 mg of fecal material was taken from the collection tube, along with 200 μl of the DNA/RNA Shield supernatant. Next, 7.78 μl of Spike-in Control I (D6320; Zymo Research, Irvine, United States) was added, containing 7.78 × 10^7^ cells of the gram-negative *Imtechella halotolerans* and gram-positive *Allobacillus halotolerans*. DNA extraction was performed according to the three peaks DNA extraction method (Quick, 2019), with the following modifications: Bead-beating was performed on a Bertin Minilys (Bertin Technologies, Montigny-le-Bretonneux, France) at 3,000 rpm. Next, the sample was split in two and divided into two 1.5 ml Eppendorf tubes for the DNA-binding step with Genomic lysis buffer (D3004-1; Zymo Research, Irvine, United States). After the DNA-binding step, the magnetic beads in both tubes were each resuspended in 50 μl DNA elution buffer (D3004-4; Zymo Research, Irvine, United States), and were recombined back into a single 1.5 ml Eppendorf tube. Finally, the elution was done on a thermoshaker at 55°C and 200 rpm for 5 min.

FQ resistant ST10 *E. coli* isolates were obtained from four broiler farms (farms A and B mentioned before, as well as from two additional farms, i.e., C, D) as described in Ringenier et al. (under review)^1^, and were grown overnight in Luria-Bertani medium at 37°C and 250 RPM shaking. Isolates were characterized using short-read (Illumina) and long-read (ONT) sequencing. For Illumina sequencing, the GenElute™ Bacterial Genomic DNA Kit from Sigma-Aldrich (Merck, Overijse, Belgium) was used according to the manufacturer's protocol, as described (Ringenier et al., under review)^1^. For ONT sequencing, DNA extraction was performed as described for the fecal metagenomic samples, with omission of the bead-beating step.

### 2.2 Nanopore sequencing and basecalling

Metagenomic nanopore sequencing libraries were generated using the ligation sequencing kit, while isolate libraries were prepared using the native barcoding kit (respectively, SQK-LSK114 and SQK-NBK114.24, Oxford Nanopore Technologies, Oxford, United Kingdom). The manufacturer's instructions were followed for all libraries. The metagenomic samples were sequenced on one MinION R10.4.1 flow cell (FLO-MIN114, Oxford Nanopore Technologies, Oxford, United Kingdom) per sample on a GridION device (Oxford Nanopore Technologies, Oxford, United Kingdom), while the isolates libraries were barcoded and combined on a single R10.4.1 flow cell. For all samples, basecalling was performed using Dorado 0.7.0 and the super high accuracy basecalling model v5 (dna_r10.4.1_e8.2_400bps_sup@v5.0.0), using the option “–modified-bases 4mC_5mC 6mA” to call DNA methylation, with methylation model versions (dna_r10.4.1_e8.2_400bps_sup@v5.0.0_4mC_5mC@v3 and dna_r10.4.1_e8.2_400bps_sup@v5.0.0_6mA@v3). For the barcoded isolate libraries, the option “–barcode-both-ends” was used in dorado for demultiplexing. Reads were filtered for Q-scores ≥ 10 and length ≥ 500 using SeqKit v2.3.0 (Shen et al., 2024).

### 2.3 Read-mapping based metagenomic analysis

Taxonomic classification of the reads was performed using Kraken2 (Wood et al., 2019) v2.1.1 with default settings to a large in-house database containing: complete, chromosome and scaffold NCBI Refseq assembly levels of Archaea, Fungi, Protozoa and Plant; complete Refseq assemblies of Bacteria and Viruses; and complete and chromosome assemblies of Animals (including *Gallus gallus*), supplemented with complete and chromosome assemblies for Plants from GenBank. The databases were accessed on January, 13th 2024 (O'Leary et al., 2016; Bogaerts et al., 2025). Next, reads assigned to Eukaryota were filtered out using the “extract_kraken_reads.py “script from KrakenTools v1.2, with parameter “-t” set to “2759,” and the “-exclude,” “-include-children” and “-fastq-output” parameters (Lu et al., 2022). The remaining reads where then mapped with KMA v1.4.15 (Clausen et al., 2018) to an in-house database consisting of archaea, fungi, plants and protozoa with complete, chromosome and scaffold assembly levels, and bacteria and viruses with complete assembly levels, obtained from the NCBI RefSeq database on February 24th 2023. The following options were used: “-bc” set to 0.7, “-ID” set to 0.0, “-proxi” set to “0.9,” and “-mem_mode,” “-bcNano,” “-ef,” “-1t1,” “-ca” enabled. KMA v1.4.15 was additionally utilized to map the non-Eukaryotic reads to the ResFinder database version 2.4.0 (Bortolaia et al., 2020), with the same options as for taxonomic mapping, excluding the “-1t1” setting for reads potentially carrying more than one ARG. ARGs were then filtered for template identity of more than 90%. Reads mapping to both a taxonomic and an ARG template were used to investigate species-ARG links (Bloemen et al., 2023), using a custom python script. Finally, the data were visualized using custom Python (v3.12.3) and R (v4.1.3) scripts. The complete workflow is available as an open-source Snakemake workflow on GitHub: https://github.com/brambloemen/FLUPOULmeta.

### 2.4 Assembly-based metagenomic analysis and detection of methylation motifs

The pipeline for metagenomic assembly, binning, and methylation analysis is also included in the GitHub repository mentioned above (https://github.com/brambloemen/FLUPOULmeta), and is further described here. The filtered non-Eukaryotic reads (produced as described in Section 2.3) were assembled using Flye v2.9.4 (Kolmogorov et al., 2020), using the options “-nano-hq -meta -i 2 -read-error 0.03.” Next, a single iteration of polishing was performed with Medaka v2.0.0, using the mini_align, inference and sequence modules with the following basecalling model: dna_r10.4.1_e8.2_400bps_sup@v5.0.0. Contigs were then binned using an ensemble of methods including: MetaBAT2 v2.15 with default options (Kang et al., 2019), graphMB v0.1.5 with the options “-assembly_type flye -vamb -minbin 250000 -mincontig 3000” (Lamurias et al., 2022) and SemiBin v2.1.0 with options “-compression=none -sequencing-type long_read -environment global” (Pan et al., 2023). The outputs from all binning methods were combined using DASTool v1.1.6 with the option “-score_threshold” set to 0 (Sieber et al., 2018). For methylation analysis including methylation-based bin improvement, the raw reads (including methylation tags) were mapped back to the assembly using Minimap2 v2.28 (Li, 2018), using options “-ax map-ont –y,” and were processed further with SAMtools v1.17 (Danecek et al., 2021). After this, a methylation pileup was made using Modkit v0.3.0. Next, NanoMotif v0.5.6 (Heidelbach et al., 2024) was used for methylation analysis and bin improvement as follows. Motif discovery was carried out using the “motif_discovery” module with the following options: “-min_motif_score” set to 0.2, “-read_level_methylation” enabled, and “-threshold_valid_coverage” set to 1. Bin inclusion was done using the “include” function of NanoMotif with the “-run_detect_contamination” option enabled. Classification of the MAGs corrected with NanoMotif was done using GTDB-Tk v2.3.2 with GTDB database release 214 (Chaumeil et al., 2022), using the command “gtdbtk classify_wf “with option “-skip_ani_screen” enabled. Completeness and contamination of MAGs were checked using the “checkm lineage_wf” command from CheckM v1.2.2 (Parks et al., 2015) with default options. The methylation output reported by NanoMotif in the “motifs-scored.tsv” file were analyzed by visualizing the methylation percentage per motif, using the R packages ComplexHeatmap v2.10.0 (Gu et al., 2016) and UMAP v0.2.10.0 (McInnes et al., 2020). Motifs were only used if they occurred in at least one contig with more than 50% methylation. For each bin, the 10 largest contigs were included, and bins were filtered for >10% completeness. NCBI's AMRFinderPlus v3.12.8 with database version 2024-05-02.2 was used to detect ARGs (Feldgarden et al., 2021), along with KMA and the ResFinder database as described in Section 2.3 for read-based analysis. For *Klebsiella pneumoniae* or *Escherichia coli* MAGs, AMRFinderPlus v3.12.8 was run separetely with the “-organism” option set to the corresponding species. AMRFinderPlus and KMA were used in the same way to detect ARGs in the reference genomes of the spike-in species (Refseq accessions NZ_CP117969.2 for *I. halotolerans* and NZ_CP117968.1 for *A. halotolerans*), detecting no ARGs. Plasmids and mobile elements were detected with an ensemble approach: First, genomad v1.8.0 (Camargo et al., 2023) was run to detect plasmid/viral sequences. Next, SCAPP v0.1.4 (Pellow et al., 2021) was used to detect remaining plasmid contigs not reported by genomad (e.g., plasmids consisting of multiple contigs that could only be detected with assembly graph information). MOB-suite v3.1.9 (Robertson et al., 2020) was additionally used to profile plasmids. Finally, mobileOG-db with the mobileOG-pl scripts (https://github.com/clb21565/mobileOG-db/tree/main commit 91ee129; Brown et al., 2022) was used to detect MGE-related genes. Proksee was used to illustrate plasmids and mobile elements (Grant et al., 2023).

### 2.5 Strain phasing and strain-level detection of resistance-associated SNPs

Longshot v1.0.0 (Edge and Bansal, 2019) was utilized to call variants with default settings (as indicated in the Floria documentation), after which Floria (Shaw et al., 2024, git commit 22fb0ab) was used to phase reads into haplotypes. The results were visualized with the script “visualize_vartigs.py” in the Floria github repository. Phased reads were then clustered into strains with floria-strainer v0.2.1 (https://github.com/maxibor/floria-strainer). Each set of clustered reads was then separately assembled using the ONT assembly method for isolates described in Section 2.6. The resulting phased assemblies were checked for quality with CheckM (Parks et al., 2015) as described in Section 2.4, and ARGs and resistance-conferring SNPs were analyzed using AMRFinderPlus as described in Section 2.4, with the additional “-organism” option set to the organism of interest to identify SNPs. Further manual inspection of SNPs was performed with SAMtools v1.17 (Danecek et al., 2021) and IGV (Thorvaldsdóttir et al., 2013).

### 2.6 Bioinformatic analysis of isolates, phylogenetic comparison and plasmid alignment

Illumina short-read sequencing data for *E. coli* isolates was generated, assembled and sequence typed as described in Ringenier et al. (under review)^1^, using a previously published STEC pipeline (v1.0, available at https://galaxy.sciensano.be; Bogaerts et al., 2021). ONT assemblies for the isolates were generated with Flye (Kolmogorov et al., 2020) and Medaka as described in Section 2.4, but without the “-meta” option in Flye, and without further binning steps. ARGs were detected as described in Section 2.4. Multi-locus sequence typing (MLST) of the metagenomic strain-phased genome and isolate assemblies was performed on the Sciensano Galaxy instance (Bogaerts et al., 2025), using the seven-gene university of Warwick scheme (Zhou et al., 2020), updated September 1st 2024. For SNP-phylogeny of the metagenomic strain-phased ST10 genome in sample A and ST10 isolate genomes, PACU v0.0.7 (Bogaerts et al., 2024) was applied as follows: First, both long- and short-read data of a single isolate (Farm A Isolate 3) were used with an in-house implemented hybrid assembly pipeline to generate a common reference genome (Bouras et al., 2024). Only the largest contig in this hybrid assembly (4.9 Mb), representing the *E. coli* chromosome, was retained as the reference genome for PACU v0.0.7 (Bogaerts et al., 2025). Next, the web-based PHASTEST (Wishart et al., 2023) was used to identify phage regions in the reference, from which a BED file was created to mask phage regions in the PACU analysis with the “-ref-bed” option. For each isolate, reads (Illumina or ONT) were then mapped to the reference genome with the “PACU_map” script (Bogaerts et al., 2024). Strain-phased reads from the metagenomic data were converted from BAM to FASTQ format with the SAMtools “fastq” command, and were then aligned to the reference genome using the approach described above. Next, the PACU pipeline was used with the reference-mapped read sets to construct a phylogeny, with phage regions masked, and other settings left at default values. To compare the metagenomically detected and linked plasmid, hybrid assemblies for two additional isolates (Farm A Isolate 1 and 2) were generated as described above for the PACU reference genome. Finally, the dnadiff command from Mummer4 v4.0.0rc1 (Marçais et al., 2018) was used to align and compare the metagenomic plasmids and the hybrid assemblies for the isolates (Farm A Isolate 1 and 2) to the reference (Farm A Isolate 3).

## 3 Results

### 3.1 Read-based resistome analysis of chicken fecal samples

**Figure 1:**
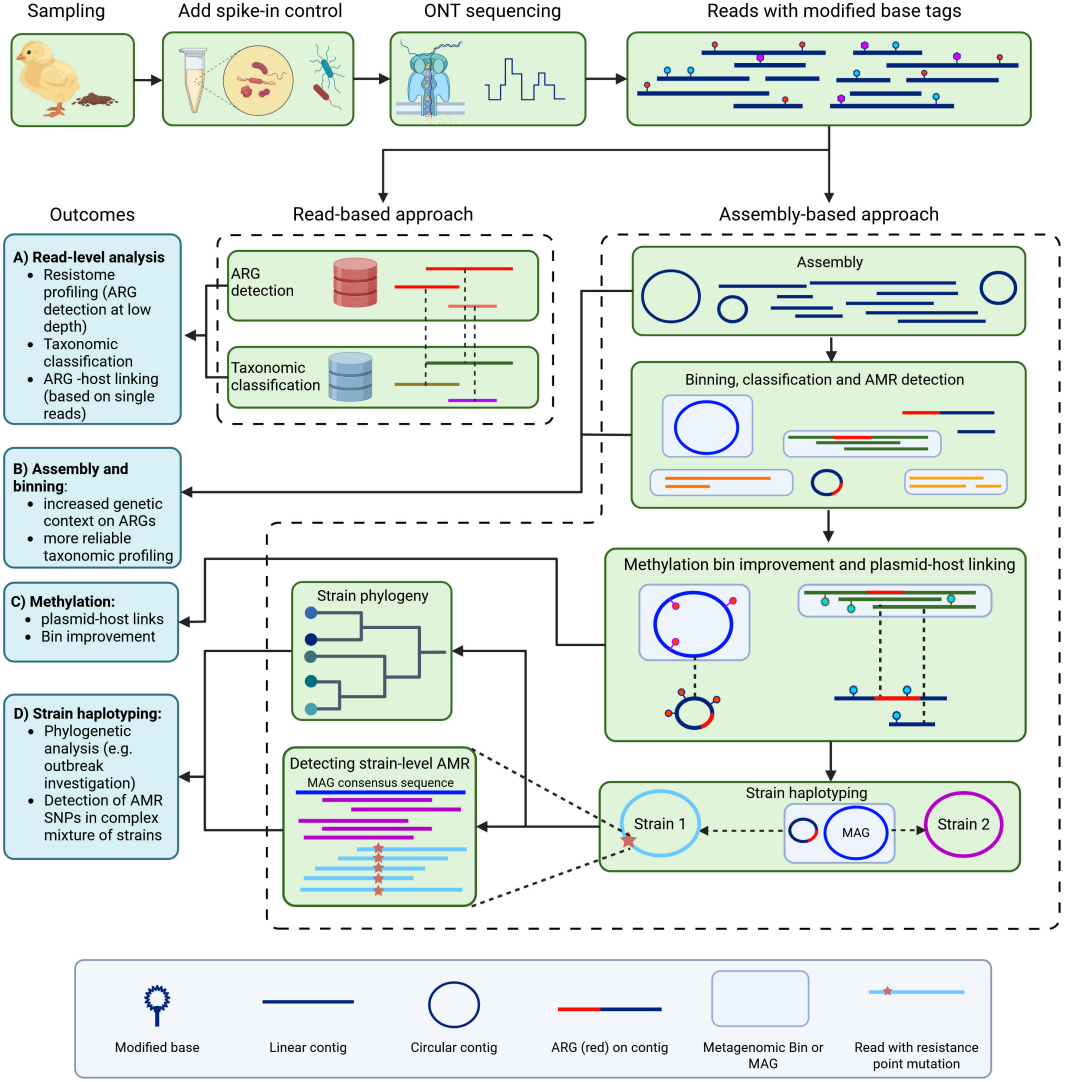
Overview of metagenomic antimicrobial esistance (AMR) investigation. We sampled chicken feces from farms, added spike-in control bacteria, and used Oxford Nanopore Technologies sequencing with methylation calling to generate long reads with methylation base tags. Antimicrobial resistance genes (ARGs) were then identified using a read-based approach **(A)**, and through metagenomic assembly and binning (assembly-based approach) **(B)**. For the read-based approach, ARG-carrying reads were also retrieved from their taxonomic alignment for host linking. For the assembly-based approach, methylation motifs were used for bin improvement and plasmid-host linking **(C)**. Strain haplotyping then allowed for phylogenomic investigation and detection of strain-level AMR, including point mutations associated with AMR, which we did for fluoroquinolone resistance **(D)**.

**Figure 2:**
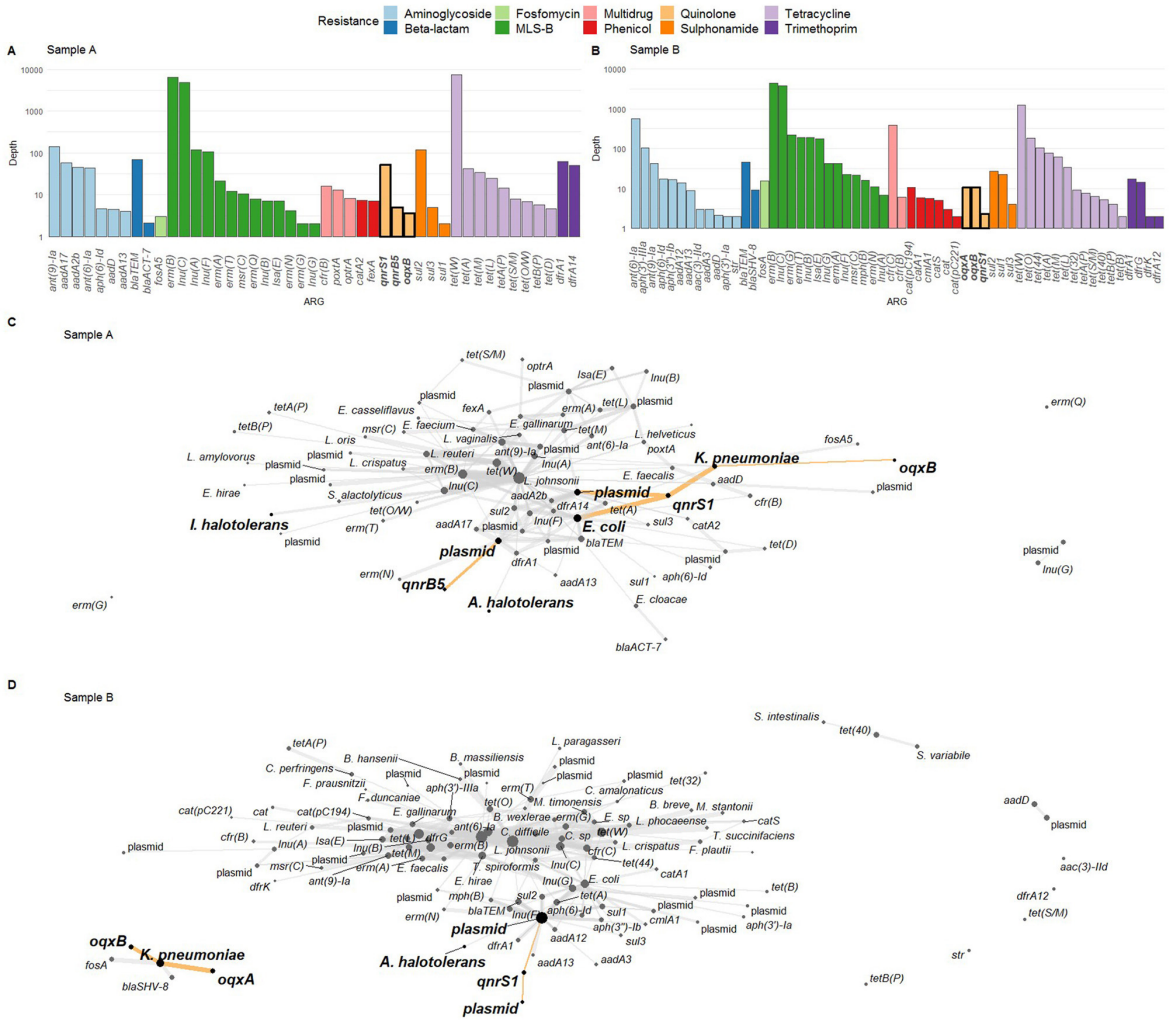
**(A, B)** Resistome profile in terms of logarithmically scaled depth of coverage per antimicrobial resistance gene (ARG) for samples A and B, respectively, with fluoroquinolone (FQ) ARGs indicated in bold on the x-axis. **(C, D)** ARG-host links as determined via ARG detection in reads and read taxonomic alignment. FQ ARGs and their hosts are highlighted with bold nodes, bold text, and highlighted edges. In **(C)**, both *I. halotolerans* and *A. halotolerans* are indicated as carrying an ARG. In **(D)**, *A. halotolerans* is reported as ARG host as well. MLS-B: macrolide, lincosamide, streptogramin-B.

To perform a resistome analysis, we collected chicken fecal samples from two farms (A and B, Supplementary Table S1), and performed ONT sequencing (Figure 1). For sample A, this yielded 14.55 Gigabases (Gb) of total DNA on 1.61 × 10^6^ reads, with an N50 of 15.03 kb. For sample B, 15.03 Gb of data was obtained on 3.14 × 10^6^ reads, with an N50 of 9.94 kb. Initially, read-based ARG detection and taxonomic host linking were performed (Figure 1A). The following FQ resistance genes were detected in sample A: *qnrS1, qnrB5*, and *oqxB*, but not the *oqxA* part of the *oqxAB* operon (Figure 2A). In sample B, the *oqxAB* operon and *qnrS1* gene were found alongside other ARGs (Figure 2B). To investigate species-ARG associations, we extracted reads that mapped to both ARGs and taxonomic templates. In sample A, the *qnrS1* gene was linked to *Klebsiella pneumoniae, Escherichia coli* and a plasmid according to taxonomic mapping (Figure 2C). The *qnrB5* gene was associated with a plasmid, while no taxonomic match was found for *oqxB*. Surprisingly, the spike-in control strains *I. halotolerans* and *A. halotolerans* were also linked to an ARG, while the reference genomes do not contain any. In sample B, the *oqxAB* operon was linked to *K. pneumoniae*, while *qnrS1* was associated to plasmids (Figure 2D). Similar to sample A, *A. halotolerans* was also reported as ARG host in sample B. Point mutations were not investigated using the read-based resistome analysis workflow described here.

### 3.2 Metagenome assembly to investigate genetic context and host species of fluoroquinolone resistance genes

To investigate the broader genetic context of the FQ ARGs, we performed an assembly-based analysis, and binned the resulting contigs (Figure 1B). In both samples, the spike-in control species were retrieved as high-quality MAGs (>90% completion and < 5% contamination), except for *I. halotolerans* in sample A, which had the lowest coverage of 4.39X and was recovered at 87.52% completion (Supplementary Table S2). Most of these MAGs consisted of multiple contigs (8 to 16 contigs, with N50 ranging from 186 kbp to 1.9 Mbp), except for *A. halotolerans* in sample B, where a single 2.7 Mbp contig was assembled.

Next, the assemblies were screened for the presence of plasmids, MGEs, and for ARGs (Figure 1B). We compared the detection of FQ resistance genes in the overall metagenome assemblies to their detection in reads (Table 1). In contrast to the multiple hosts reported for *qnrS1* in sample A, it was found exclusively on a single conjugative IncFII, MOBP plasmid in the assembly, which carried various other ARGs such as *blaLAP-2* and *tet(A)* (Supplementary Figure S1). However, this plasmid could not be attributed to a metagenomic bin. For both *E. coli* and *K. pneumoniae*, MAGs were obtained, although the *K. pneumoniae* MAG was of low completeness (Table 1). The *E. coli* MAG was highly complete, but the high strain heterogeneity indicates that the MAG represents a collapse of multiple strains. The *qnrB5* and *oqxB* genes observed in the read-based approach were not detected in the metagenomic assembly of sample A. In sample B, the *oqxAB* operon was located on a high quality *K. pneumoniae* MAG, supporting the read-based analysis. Additionally, the operon was found in proximity to multiple mobile element related genes, as determined by mobileOGdb (Supplementary Figure S2). The *qnrS1* gene was not detected in the sample B assembly. In both samples, no FQ resistance-associated SNPs were found in the *E. coli* and *K. pneumoniae* bins (Table 1). Finally, in both samples no ARGs were detected in MAGs obtained for the spike-in species *A. halotolerans* and *I. halotolerans*, in contrast to the read-based approach where these species were indicated as ARG hosts.

**Table 1:** Overview of fluoroquinolone (FQ) resistance genes.

**Sample**	**FQ resistance gene/mutation**	**Detected in assembly and reads**	**Classification**	Depth of coverage^*^	**CheckM completeness (%)**	**CheckM contamination (%)**	**CheckM strain heterogeneity (%)**
A	*qnrS1*	Yes	IncFII, MOBP plasmid	36.20	n.a.	n.a.	n.a.
	*qnrB5*	Only in reads	Not detected in assembly, but reads classified as *E. coli* plasmid.	4.00	n.a.	n.a.	n.a.
	no FQ ARG/mutation found in bin	/	*Escherichia coli*	32.3	99.97	0.99	77.78
	no FQ ARG/mutation found in bin	/	*Klebsiella pneumoniae*	3.68	12.07	0.00	0.00
	*oqxB*	Only in reads	n.a.	2.57	n.a.	n.a.	n.a.
B	*oqxAB*	Yes	*Klebsiella pneumoniae*	8.71	98.79	0.04	0.00
	*qnrS1*	Only in reads	n.a.	1.29	n.a.	n.a.	n.a.

If these were detected in the assembly, metrics for the bin or contig are given. Additionally, if read-level analysis (cfr. Figure 1) identified a species as a potential FQ ARG host, the bin representing these hosts is included here as well. ^*^Depth of coverage: For bins (with or without ARGs) with multiple contigs, the depth of coverage is calculated as the weighted mean of contig depths, weighted by contig length. If detected on an unbinned contig (e.g., qnrS1 in sample A), the depth of the contig is given. If an ARG was not detected in the assembly but only in reads, the coverage depth of the ARG template is given. n.a., not applicable; /, not detected.

### 3.3 Detection of DNA methylation to reveal plasmid-host associations

**Figure 3:**
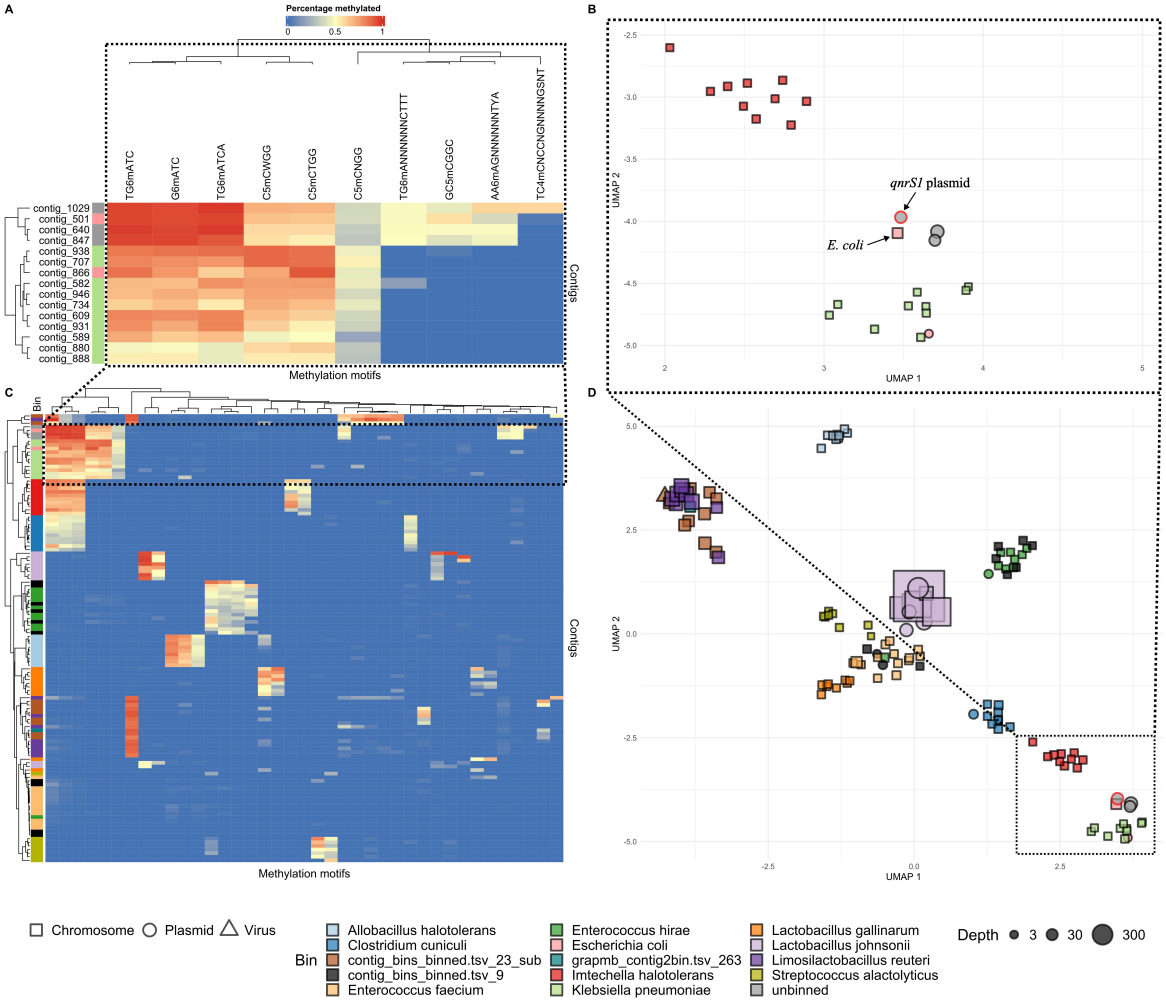
Contig methylation profiles in sample A, visualized in hierarchically clustered heatmaps **(A, C)** and UMAP plots **(B, D)**. Colors next to the rows (representing contigs) in **(A)** and **(C)** indicate the metagenomic bins as determined by the Nanomotif-refined binning method, while columns represent methylation motifs. Cell colors represent methylation percentage. In the UMAP plots (B, D), contigs are shaped according to their genomad classification, and colored by bin [cfr **(A, C)**]. In all plots, motifs were only included if they were at least 50% methylated in at least a single contig. Bins with completeness <10% are excluded, and for each bin the largest 10 contigs are shown. The upper plots **(A, B)** focus on the unbinned plasmids (contig 847, 1,029, and 640, gray) and the bins with the most similar methylation profiles to these plasmid: *E. coli* (light red) and *K. pneumoniae* (green). The *qnrS1-*carrying plasmid [contig 847 in **(A)** and indicated in **(B)**] most closely resembles the *E. coli* genome in terms of methylation [contig 501 in **(A)**, indicated in **(B)**]. Contig 866 (light red) is binned as *E. coli*, but clusters more closely with *K. pneumoniae* contigs in **(A, B)**. The lower plots **(C, D)** show the overall methylation profiles of the metagenomic assembly. A legend shows al bins by color. Some bins (e.g., contig_bins_binned.tsv_23_sub) were >10% complete, but could not be classified to species or genus level using gtdb-tk.

Since no bacterial host could be determined for the *qnrS1*-carrying plasmid in sample A, we investigated the use of methylation motifs to associate it to a potential host (Figures 1C, 3). Initially, neither the metagenomic pipeline nor the NanoMotif “include_contig” command assigned the *qnrS1*-carrying plasmid to any bin. However, visualization of methylation motifs indicated that the methylation patterns of three unbinned plasmids, among which the *qnrS1*-carrying plasmid, resembled the pattern of the *E. coli* and the *K. pneumoniae* bins. Still, the motifs TG6mANNNNNNCTTT (and AA6mAGNNNNNNTYA, representing its reverse complement) and GC5mCGGC were methylated in the *qnrS1*-carrying plasmid and in *E. coli*, but not in *K. pneumoniae*, indicating the plasmid likely belongs to *E. coli* (Figure 3A). In contrast, methylation signatures for a plasmid contig binned as *E. coli* (contig 866, Figure 3A) clustered more closely with the *K. pneumoniae* bin, as it was not methylated for the motifs mentioned above. Furthermore, the *K. pneumoniae* contigs and contig 866 demonstrated more variation in their degree of methylation of the detected motifs compared to the *E. coli* chromosomal contig and the three unbinned plasmids indicated in Figures 3A, B, complicating methylation-based analysis. Similarly, some other bins were not clearly separated by their methylation profiles, as demonstrated by the interleaved rows for some bins in Figure 3C (e.g., some unclassified bins and the *Enterococcus hirae, Enterococcus faecium*, or *Limosilactobacillus reuteri* bins). However, we observed that for other bins, the visualized methylation profiles did correspond to the output of the binning pipeline. This includes the internal control species *I. halotolerans* and *A. halotolerans* (Figures 3C, D).

### 3.4 Long-read haplotyping uncovers fluoroquinolone resistance SNPs and enables MLST

As SNPs in genes encoding target enzymes are an important mechanism of FQ resistance (Kherroubi et al., 2024), the MAGs in both samples were screened, but initially no FQ resistance SNPs were found. However, as MAGs often represent a species-level consensus, they can mask low-frequency FQ resistant strains. Therefore, we performed a haplotype-phasing analysis (Figure 1D), focused on FQ resistance for several MAGs that were indicated as having high strain heterogeneity (Table 2). In sample A, two phased assemblies were produced for the *E. coli* MAG: a first (phased 1) detected at a coverage of 31.3X, with high completeness and reduced contamination than the original MAG; and a second (phased 2) at a lower coverage of 11.9X, with lower completeness and higher contamination than the original MAG (Table 2, Supplementary Figure S3). However, no FQ resistance SNPs were found in either phased assembly in sample A. Using MLST, the phased 1 assembly could be identified as belonging to the ST10 sequence type. Interestingly, some FQ resistant *E. coli* isolates obtained from four farms, including A and B, were also typed as ST10 by MLST (Ringenier et al., under review)^1^. In contrast, no sequence type could be determined for the phased 2 assembly. For the *E. coli* MAG in sample B, three phased assemblies were generated with varying coverages and CheckM quality measures. One of these (phased 2) contained the S83L mutation in *gyrA*, while the other two (phased 1 and 3) did not. However, a high level of contamination and strain heterogeneity was still observed for the phased 2 assembly, which inhibited MLST analysis. Inspection of the phased reads demonstrated that the majority of reads in the phased 2 set contained the mutation, while a smaller proportion did not (Supplementary Figure S5). In the phased 1 assembly, no FQ-resistance associated mutation was observed. In the phased 3 assembly, no FQ resistance-related SNPs were detected. However, as the assembly was only 53.8% complete and had high strain heterogeneity, it is possible that some FQ resistance-related SNPs were not detected. None of the farm B phased assemblies could be sequence typed.

**Table 2:** Strain haplotype phasing results for the investigated MAGs, along with fluoroquinolone (FQ) resistance-related point mutations.

**Sample**	**FQ resistance mutation**	**Classification**	Depth of coverage^*^	**CheckM completeness (%)**	**CheckM contamination (%)**	**CheckM strain heterogeneity (%)**
A	/	*Escherichia coli*—MAG	32.3	99.97	0.99	77.78
A	/	*Escherichia coli*—phased 1	31.3	99.97	0.04	0.00
A	/	*Escherichia coli*—phased 2	11.9	68.65	13.79	100.00
B	/	*Escherichia coli*—MAG	39.5	95.21	1.53	85.71
B	/	*Escherichia coli*—phased 1	25.6	91.63	0.30	100.00
B	*gyrA* S83L	*Escherichia coli*—phased 2	18.3	99.87	7.75	98.20
B	/	*Escherichia coli*—phased 3	7.39	53.80	1.60	57.14

Both the original MAG and the strain-phased assemblies are shown.^*^Coverage depth was calculated as the mean depth over all contigs, weighted by contig length. /, not detected.

### 3.5 Phylogenomic comparison of metagenomics data and isolates provide further evidence for plasmid-host link

**Figure 4:**
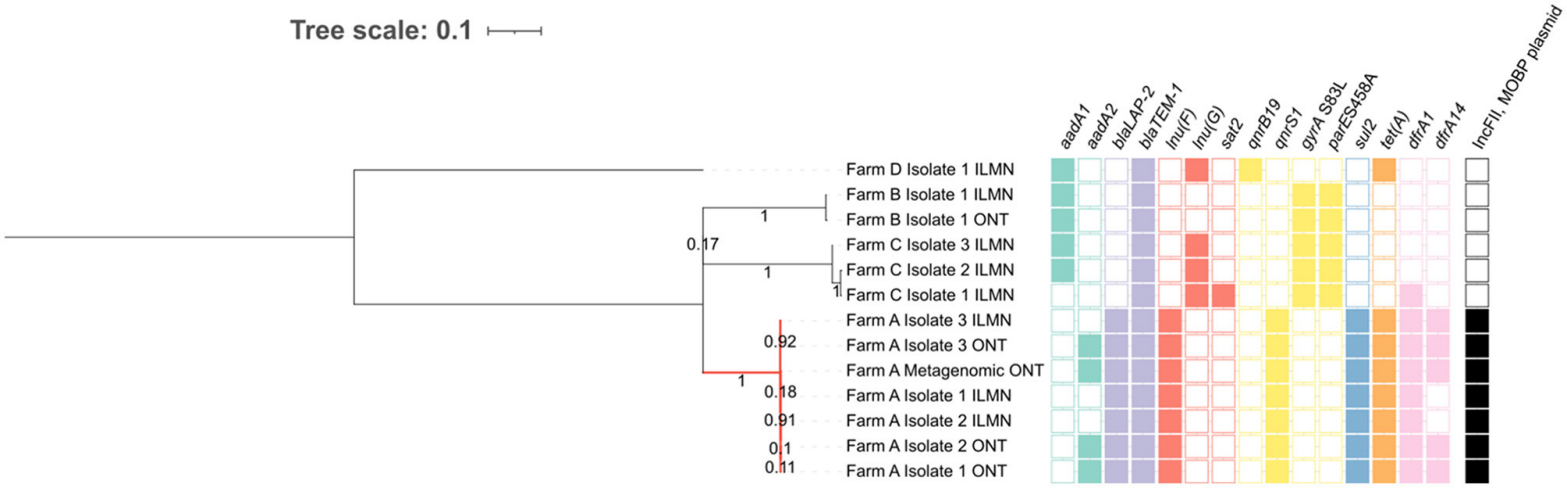
SNP-based phylogeny of *E. coli* ST10 isolates obtained from multiple farms and the sample A *E. coli* MAG. As reference sequence, a hybrid assembly of Farm A Isolate 3 was used, from which plasmids were removed. All Farm A isolates (red branch) cluster closely together, contain a similar antimicrobial resistance gene (ARG) profile and carry an IncFII, MOBP plasmid with the *qnrS1* gene. All ARGs in the farm A isolates were carried on plasmids. The ILMN: Illumina short reads, ONT: Oxford Nanopore Technologies long reads. Branch length and three scale represent average number of substitutions per site. The numbers on the branches represent bootstrap values.

To confirm that the *qnrS1*-carrying plasmid in sample A originated from an *E. coli* strain, as suggested by the metagenomic DNA methylation analysis (cf. 3.3) we performed two additional analyses: We sought whether an isolate representing the metagenomic strain could be found, and next if this isolate indeed carried the *qnrS1-*carrying IncFII, MOBP plasmid. To accomplish this, we phylogenomically compared the metagenomic *E. coli* strain to several previously isolated ST10 strains, some of which carried an IncFII, MOBP plasmid and the *qnrS1* gene (Figure 4). A hybrid assembly using ONT long-read and short-read data of one of the isolates (Farm A isolate 3) was used as the reference genome, from which plasmids were removed. Some other isolates were re-sequenced with ONT, and these data were also included in the phylogenomic analysis. The resulting phylogenetic tree demonstrates that the *qnrS1*-carrying farm A isolates are (nearly) identical to the *E*. coli phased 1 strain (Figure 4). Specifically, the metagenomically detected strain differs by 0 SNPs to isolates 1 and 2 from farm A, and by 3 SNPs to isolate 3 from farm A (Supplementary Table S3), indicating that they all represent the same strain. Additionally, long-read assemblies of the farm A isolates contained the same ARGs as the metagenomically phased strain, and similarly no FQ resistance-conferring SNPs. However, the short-read assemblies were missing several ARGs compared to the (metagenomic) long-read assemblies (Figure 4). In both the metagenomic assembly and the isolate hybrid assembly used as reference, these genes were situated on an IncFII, MOBP plasmid, and were hence not included in phylogenetic analysis.

Next, to confirm that the *qnrS1*-carrying IncFII, MOBP plasmid in the sample A MAG was identical to the IncFII, MOBP plasmids in the farm A isolates, we aligned the metagenomically detected plasmids to the plasmids in the reference sequence (the hybrid assembly of farm A isolate 3, Table 3). Additionally, IncFII, MOBP plasmids from the other two farm A isolates (1 and 2) were also aligned to the reference. Both the IncFII, MOBP plasmid from the metagenomics data, as well as those from the hybrid assemblies were highly similar to the reference plasmid, with coverage of 100% and identity of at least 99.96% (Table 3). Finally, we further investigated the complete plasmid content of the farm A isolates to confirm whether the detection of the other plasmids and their methylation-based association to *E. coli* in the sample A metagenomic data (cfr. 3.3) was correct (Table 3). Aside from the IncFII, MOBP plasmid mentioned earlier, a second and third plasmid, contig 2 and 4 in the reference genome, were also found in all isolates. A fourth plasmid, contig 7 in the reference, was detected in all isolates, but not in the metagenomic assembly. In the reference and isolate 2 it represented a small plasmid of 6,446 bp, but in isolate 1 it was detected as a 3,223 bp plasmid. Interestingly, the plasmid contig that was binned with *E. coli* in the sample A metagenomic results (contig 866 on Figure 3A) did not have a counterpart in the plasmids from the reference genome. However, it partially aligned to the *E. coli* chromosome in the reference, but only with 21.91% of its sequence (Supplementary Table S4). In summary, from the four plasmids consistently detected in the isolates, we detected three in the sample A metagenomic assembly and correctly attributed them to *E. coli* based on further methylation analysis (cfr 3.3). In contrast, one plasmid associated to the *E. coli* MAG by the binning pipeline was not observed in the isolates.

**Table 3:** Alignment results of the metagenomic plasmids and the plasmids from isolate hybrid assemblies to plasmids in the Farm A Isolate 3 reference.

**Sample**	**Identity (%)**	**Reference length**	**Query length**	**Reference coverage**	**Query coverage**	**Reference contig**	**Query contig**
Farm A metagenomic	99.99	326,267	326,266	100.00	100.00	contig_2	contig_640
	99.42	108,180	106,863	98.32	99.56	contig_4	contig_1029
	**99.99**	**81,767**	**81,767**	**100.00**	**100.00**	**contig_9**	**contig_847**
Farm A Isolate 1 hybrid assembly	99.99	326,267	326,268	100.00	100.00	contig_2	contig_6
	98.98	108,180	10,8217	99.02	98.99	contig_4	contig_1
	100.00	6446	3,223	50.00	100.00	contig_7	contig_13
	**99.99**	**81,767**	**8,1767**	**100.00**	**100.00**	**contig_9**	**contig_2**
Farm A Isolate 2 hybrid assembly	99.99	326,267	325,905	99.89	100.00	contig_2	contig_9
	99.97	108,180	108,165	100.00	100.00	contig_4	contig_4
	100.00	6,446	6,446	100.00	100.00	contig_7	contig_8
	**99.96**	**81,767**	**81,733**	**100.00**	**100.00**	**contig_9**	**contig_5**

Alignments of the qnrS1-carrying plasmid are highlighted in bold.

## 4 Discussion

### 4.1 Comparison of ARG taxonomic host detection from long reads and long-read assemblies

Long-read metagenomics holds great promise as a novel tool to detect and monitor AMR. It can complement current methods by providing a fast and comprehensive approach to detect and investigate the genetic factors behind AMR occurrence and spread. In this study, we applied recent developments in Oxford nanopore sequencing to overcome some of the key challenges in metagenomics for AMR surveillance. Specifically, we focus on attributing ARGs and plasmids to their bacterial hosts and resolving strain-level variation associated to AMR, using FQ resistance in poultry as a case study.

Apart from detecting ARGs in metagenomic data, it is also crucial to determine their taxonomic origins and genetic surroundings. However, short-read sequencing often fails to identify sufficient genomic context to do so (Abramova et al., 2024). Here, we used long reads to address this issue, and investigated FQ ARGs and their hosts with both read-based and assembly-based approaches. With read-based profiling, several FQ resistance genes were found that went undetected in assemblies, such as *qnrB5* and *oqxB* in sample A and *qnrS1* in sample B. However, these were all observed with low read counts, and their bacterial host reported by the read-based approach could not be confirmed by the assembly-based approach. Additionally, the low sequencing coverage of these ARGs likely impeded their assembly into larger contigs, explaining why they were not detected in the assemblies (Yorki et al., 2023). For example, detection of *oqxB* but not *oqxA* in sample A reads could indicate incomplete coverage of the *oqxB* surroundings, as these normally form a single operon (Li et al., 2019).

In contrast, FQ ARGs retrieved by both read- and assembly-based approaches were found at higher read counts than those exclusively observed in reads. For example, in the case of *oqxAB* in sample B, both analysis methods corresponded, and identified *K. pneumoniae* as the host. In sample A however, read-based ARG-host linking attributed the *qnrS1* gene to multiple host replicons, while it was unambiguously situated on a single plasmid in the metagenomic assembly. Moreover, read-based analysis identified *A. halotolerans* and *I. halotolerans* as carrying an ARG, while their respective MAGs did not contain any. The latter observation corresponds to the reference genomes, and illustrates how spike-in controls can be used to assess metagenomic results (Yorki et al., 2023). Furthermore, the assembled *qnrS1-*carrying plasmid in sample A contained several other types of ARGs, illustrating the need for sufficient genetic context to detect mechanisms of AMR co-selection and multi-drug resistance.

We also compared the metagenomically detected *E. coli* strain in sample A to isolates of the same strain, revealing that metagenomics retrieved three plasmids also found in the isolates, among which the *qnrS1*-carrying plasmid. When comparing the plasmid assemblies, the data from isolates and metagenomics corresponded well. However, a fourth small plasmid which was present in all isolates was not found in the metagenomic assembly, and differed in length between the isolate assemblies. This could be explained by the finding by Johnson et al. (2023) that long-read assemblers struggle with small plasmids.

Taken together, our analysis of both read- and assembly-based approaches highlight that although long reads can capture ARGs within their genetic context, determining their bacterial hosts directly from reads suffers from low precision, especially when carried on plasmids (Bloemen et al., 2023; Lou et al., 2024). On the other hand, assembly enables more accurate linking of ARGs to a replicon, but can be less sensitive at recovering ARGs at low coverages, and struggles with small plasmids (Johnson et al., 2023; Yorki et al., 2023). Novel methods exploiting the benefits of both methods, such as Argo (which was released after finalization of our study), could be tested in the future to allow for rapid, sensitive and precise ARG detection (Chen et al., 2025).

### 4.2 Methylation-based plasmid-host attribution

Despite enhancing recovery of MGEs and ARGs compared to short-read methods, long-read metagenomics still faces difficulties in associating them to a bacterial host, especially for extrachromosomal elements such as plasmids. In our case, a plasmid carrying *qnrS1* in sample A could be recovered, but was not attributed to a host species by current binning methods. We addressed this problem by applying DNA methylation calling with Oxford nanopore sequencing and methylation binning with NanoMotif (Heidelbach et al., 2024). On its own, the bin improvement module of NanoMotif did not place the plasmid in the *E. coli* MAG. However, closer manual inspection of the methylation patterns uncovered high similarity between the *qnrS1*-carrying plasmid and the *E. coli* MAG, indicating that the plasmid most likely belonged to *E. coli*. Conversely, methylation and coverage data of another plasmid (contig 866) that was binned as *E. coli* showed that it was possibly incorrectly binned. Both of these findings were supported by isolate data where the *qnrS1*-plasmid was found, and the other plasmid was not observed.

The inability of NanoMotif to correctly bin these plasmids could have multiple causes. First, erroneous basecalling or false positive methylation calls near methylated sites complicate correct methylation motif calling (Kulkarni et al., 2024; Galeone et al., 2025). Second, more complex motifs require higher coverage and motif frequencies for correct detection, which can be challenging in smaller contigs such as plasmids (Heidelbach et al., 2024). A third issue is specific to plasmids, as some have undergone selection for lower numbers of restriction-modification recognition sites (Shaw et al., 2023; Dimitriu et al., 2024). Therefore, our example indicates that methylation data alone can be insufficient to identify a plasmid host, especially when multiple closely related species with conserved methyltransferases are present in the same sample. As an example, the *E. hirae* and *L. reuteri* bins detected in sample A presented similar methylation patterns to other, unclassified bins. Conversely, the spike-in species *I. halotolerans* and *A. halotolerans* had more distinct methylation profiles, and could be clearly separated from all other bins, again highlighting the benefit of adding spike-in controls. Increased coverage of the incompletely separated bins, for example by using ONT adaptive sampling (which allows to accept or reject DNA molecules for sequencing based on analysis of an initial part; Martin et al., 2022), could have better distinguished their genomes by uncovering more DNA methylation motifs. Also, higher coverage and more accurate methylation calling models would likely improve methylation detection and the methylation-assisted binning process in general, as NanoMotif requires sufficient coverage and motif occurrences for reliable motif detection, depending on the type of motif (Heidelbach et al., 2024; Galeone et al., 2025). In our case-study, methylation could be used to point out a likely plasmid-host link, but isolate data were needed to reduce uncertainty. Other novel culture-independent technologies such as Hi-C or single-cell sequencing methods could likely overcome many of the limitations of methylation-based plasmid binning, but are more costly (Brito, 2021; Yang et al., 2025). In contrast, DNA methylation can provide valuable insights in a more cost-effective manner, despite the lower resolution in determining plasmid-host links.

### 4.3 Strain haplotyping reveals strain-level resistance-associated point mutations

Although long-read metagenomic assembly performs well in recovering MAGs, these often represent a species-level consensus, concealing strain-level variation such as resistance-associated SNPs, and inhibiting strain-level phylogenomic analysis. Therefore, we further investigated MAGs suspected of representing multiple subpopulations with a strain-haplotyping approach. In sample B, this uncovered a FQ resistance point mutation that was suppressed at the MAG level. In sample A, strain-level phylogenomic comparison related the metagenomic data to nearly identical isolates obtained from the same farm. However, many of the resulting phased assemblies in both samples remained incomplete or contaminated, or represented multiple strains. This was especially the case at low coverages, indicating insufficient strain separation. Novel ONT adaptive sampling approaches such as BOSS-RUNS could enhance strain haplotyping by enriching for regions with low coverage or high variation (Weilguny et al., 2023). This could improve the strain-level resolution of metagenomics, allowing for applications beyond AMR surveillance, such as outbreak investigation, e.g., when no isolate can be obtained or in time-critical situations (Buytaers et al., 2021).

## 5 Conclusion

In conclusion, we applied new developments in long-read metagenomic data analysis to address several current limitations in metagenomic AMR surveillance. While we analyzed only two samples, these provided sufficient examples to illustrate these limitations and how to overcome them. We demonstrated that read-based approaches are complementary to assembly-based approaches, with the former detecting ARGs at lower sequencing depths, while the latter can better determine their bacterial hosts. We also highlighted the value of DNA methylation detection for AMR surveillance, as this allowed association of a plasmid with its host species. Furthermore, we demonstrated the power of strain-resolved phylogenomic comparison to link metagenomically detected strains to related isolates, and to perform strain-level analysis to unveil resistance-conferring SNPs. However, several challenges remain: DNA methylation might be unable to differentiate organisms with similar methylation profiles, and insufficient coverage can limit detection of DNA methylation motifs and low frequency SNPs, inhibiting both plasmid-host association and strain deconvolution. Future work could exploit ongoing improvements in nanopore sequencing such as increases in throughput, novel ONT adaptive sampling strategies, and improved methylation calling to address these issues, enabling deeper new insights in the occurrence, spread and evolution of AMR.

## Data Availability

The original contributions presented in the study are publicly available. This data can be found here: https://www.ebi.ac.uk/ena, PRJEB86979.

## Footnotes

^1^Ringenier, M., Boyen, F., De Keersmaecker, S. C. J., Gand, M., Vanneste, K., Bogaerts, B., et al. (under review). Continued presence of fluoroquinolone resistance in *Escherichia coli* in broilers: mapping the spread (under review).
