# Supplementary material for: Overcoming challenges in metagenomic AMR surveillance with nanopore sequencing: a case study on fluoroquinolone resistance
Source: Front Microbiol. 2025 Jul 23;16:1614301. doi: 10.3389/fmicb.2025.1614301 (PMC12342191; doi:10.3389/fmicb.2025.1614301)
Supplement: Supplementary file 1 [file Supplementary_file_1.docx]

Supplementary Material

| **Sample** | **Farm** | **Accession** |
| --- | --- | --- |
| A | A | ERS23806082 |
| B | B | ERS23806087 |

Table S1: ENA accessions to sample metadata for the metagenomic data for each sample used

| **Sample** | **Classification** | **Number of contigs** | **Contig N50** | **Depth of coverage*** | **CheckM Completeness (%)** | **CheckM Contamination (%)** | **CheckM strain heterogeneity (%)** |
| --- | --- | --- | --- | --- | --- | --- | --- |
| A | *Allobacillus halotolerans* | 8 | 440229 | 5.81 | 96.62 | 2.00 | 66.67 |
| A | *Imtechella halotolerans* | 16 | 186039 | 4.39 | 87.52 | 0.33 | 0.00 |
| B | *Allobacillus halotolerans* | 1 | 2700306 | 11.69 | 98.67 | 0.67 | 0.00 |
| B | *Imtechella halotolerans* | 8 | 1914584 | 15.11 | 98.73 | 2.41 | 10.00 |

Table S2: Assembly characteristics and depth of coverage of Spike-in control I species


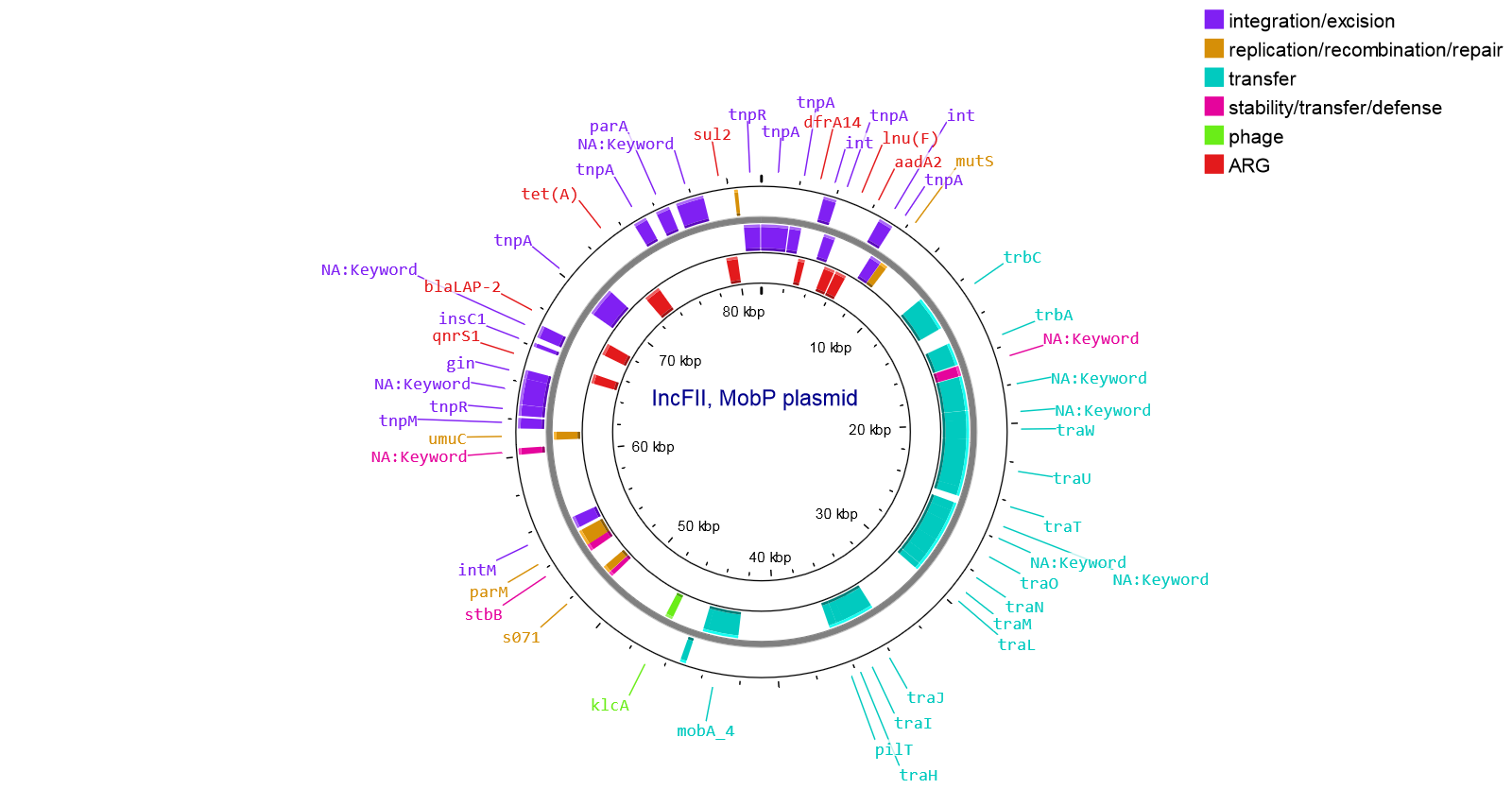
Figure S1: IncFII, MobP plasmid carrying qnrS1, generated using proksee (Grant et al., 2023)


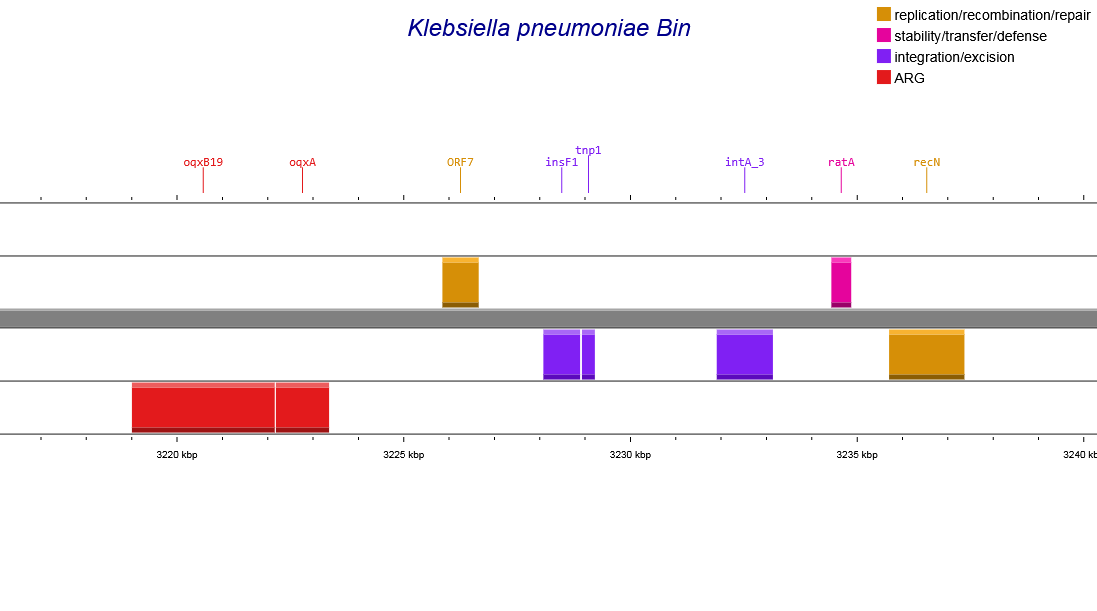
Figure S2: oqxAB genes detected in K*. pneumoniae* MAG alongside various mobile element related genes detected using mobileOGdb.


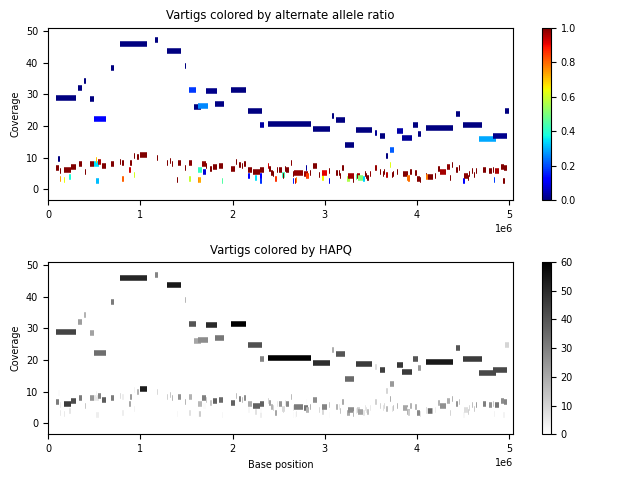


Figure S3: Floria strain phasing results on the *E. coli* MAG detected in sample A, generated using the script visualize_vartigs.py included in the Floria github repository. The plots visualize the vartigs (i.e. consensus sequences of SNPs for a given cluster of reads) by alternate allele ratio (top plot), or by haplotyping quality (bottom plot), as determined by Floria (Shaw et al., 2024).


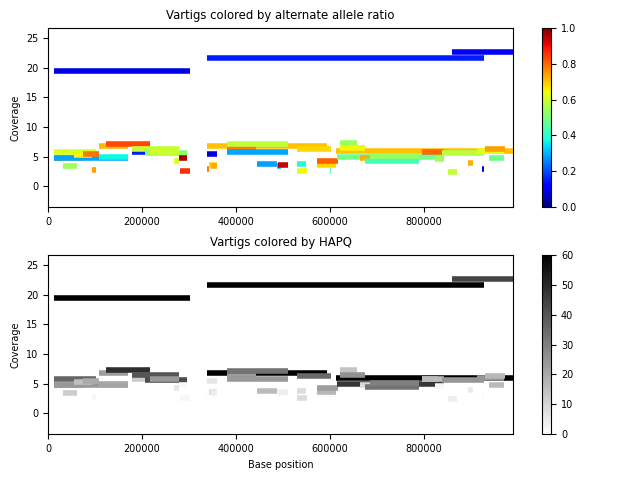


Figure S4: Floria strain phasing results on the longest contig retrieved for *E. coli* in sample B, generated using the script visualize_vartigs.py included in the Floria github repository. The plots visualize the vartigs (i.e. consensus sequences of SNPs for a given cluster of reads) by alternate allele ratio (top plot), or by haplotyping quality (bottom plot), as determined by Floria.


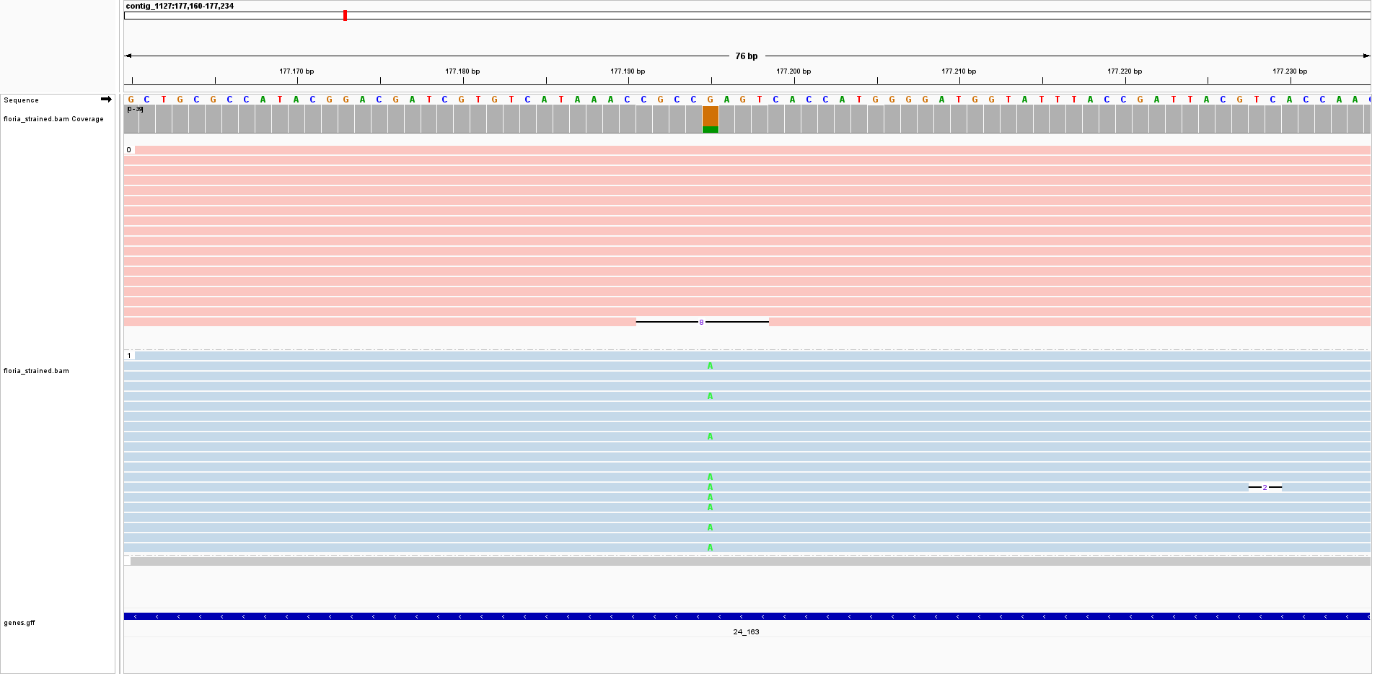
Figure S5: Phased reads indicating the gyrA_S83L point mutation which confers quinolone resistance in one of the phased set of reads.

|  | **Farm C Isolate 2 ILMN** | **Farm C Isolate 1 ILMN** | **Farm C Isolate 3 ILMN** | **Farm B Isolate 1 ILMN** | **Farm B Isolate 1 ONT** | **Farm A Metagenomic ONT** | **Farm A Isolate 1 ILMN** | **Farm A Isolate 2 ILMN** | **Farm A Isolate 2 ONT** | **Farm A Isolate 1 ONT** | **Farm A Isolate 3 ILMN** | **Farm A Isolate 3 ONT** | **Farm D Isolate 1 ILMN** |
| --- | --- | --- | --- | --- | --- | --- | --- | --- | --- | --- | --- | --- | --- |
| **Farm C Isolate 2 ILMN** | 0 | 12 | 49 | 931 | 934 | 828 | 828 | 828 | 828 | 828 | 827 | 827 | 1897 |
| **Farm C Isolate 1 ILMN** | 12 | 0 | 47 | 929 | 930 | 826 | 826 | 826 | 826 | 826 | 825 | 825 | 1897 |
| **Farm C Isolate 3 ILMN** | 49 | 47 | 0 | 916 | 919 | 785 | 785 | 785 | 785 | 785 | 784 | 784 | 1886 |
| **Farm B Isolate 1 ILMN** | 931 | 929 | 916 | 0 | 9 | 767 | 767 | 767 | 767 | 767 | 766 | 766 | 1960 |
| **Farm B Isolate 1 ONT** | 934 | 930 | 919 | 9 | 0 | 772 | 772 | 772 | 772 | 772 | 771 | 771 | 1963 |
| **Farm A Metagenomic ONT** | 828 | 826 | 785 | 767 | 772 | 0 | 0 | 0 | 0 | 0 | 3 | 3 | 1856 |
| **Farm A Isolate 1 ILMN** | 828 | 826 | 785 | 767 | 772 | 0 | 0 | 0 | 0 | 0 | 3 | 3 | 1856 |
| **Farm A Isolate 2 ILMN** | 828 | 826 | 785 | 767 | 772 | 0 | 0 | 0 | 0 | 0 | 3 | 3 | 1856 |
| **Farm A Isolate 2 ONT** | 828 | 826 | 785 | 767 | 772 | 0 | 0 | 0 | 0 | 0 | 3 | 3 | 1856 |
| **Farm A Isolate 1 ONT** | 828 | 826 | 785 | 767 | 772 | 0 | 0 | 0 | 0 | 0 | 3 | 3 | 1856 |
| **Farm A Isolate 3 ILMN** | 827 | 825 | 784 | 766 | 771 | 3 | 3 | 3 | 3 | 3 | 0 | 0 | 1855 |
| **Farm A Isolate 3 ONT** | 827 | 825 | 784 | 766 | 771 | 3 | 3 | 3 | 3 | 3 | 0 | 0 | 1855 |
| **Farm D Isolate 1 ILMN** | 1897 | 1897 | 1886 | 1960 | 1963 | 1856 | 1856 | 1856 | 1856 | 1856 | 1855 | 1855 | 0 |

Table S3: SNP distances of the ST10 E. coli MAG to ST10 isolates from various farms

Table S4: pair-wise K-mer distances of the qnrS1-carrying plasmid belonging to MOB-suite cluster AA966 from the metagenomic data compared to plasmids from the same cluster obtained in isolate sequencing.

|  | **Farm A Isolate 1 ILMN** | **Farm A Isolate 2 ILMN** | **Farm A Isolate 3 ILMN** | **Farm A Isolate 3 ONT** | **Farm A Isolate 1 ONT** | **Farm A Isolate 2 ONT** | **Farm A Metagenomic ONT** |
| --- | --- | --- | --- | --- | --- | --- | --- |
| **Farm A Isolate 1 ILMN** | 1000 | 1000 | 838 | 799 | 799 | 799 | 800 |
| **Farm A Isolate 2 ILMN** | 1000 | 1000 | 838 | 799 | 799 | 799 | 800 |
| **Farm A Isolate 3 ILMN** | 838 | 838 | 1000 | 954 | 954 | 954 | 953 |
| **Farm A Isolate 3 ONT** | 799 | 799 | 954 | 1000 | 1000 | 998 | 997 |
| **Farm A Isolate 1 ONT** | 799 | 799 | 954 | 1000 | 1000 | 998 | 997 |
| **Farm A Isolate 2 ONT** | 799 | 799 | 954 | 998 | 998 | 1000 | 999 |
| **Farm A Metagenomic ONT** | 800 | 800 | 953 | 997 | 997 | 999 | 1000 |

References

Grant, J. R., Enns, E., Marinier, E., Mandal, A., Herman, E. K., Chen, C., et al. (2023). Proksee: in-depth characterization and visualization of bacterial genomes. *Nucleic Acids Research* 51, W484–W492. doi: 10.1093/nar/gkad326

Shaw, J., Gounot, J.-S., Chen, H., Nagarajan, N., and Yu, Y. W. (2024). Floria: fast and accurate strain haplotyping in metagenomes. *Bioinformatics* 40, i30–i38. doi: 10.1093/bioinformatics/btae252
